# Compensatory mutations are associated with increased *in vitro* growth in resistant clinical samples of *Mycobacterium tuberculosis*

**DOI:** 10.1099/mgen.0.001187

**Published:** 2024-02-05

**Authors:** Viktoria M. Brunner, Philip W. Fowler

**Affiliations:** ^1^​ Nuffield Department of Medicine, University of Oxford, Oxford, UK; ^2^​ National Institute of Health Research Oxford Biomedical Research Centre, John Radcliffe Hospital, Headley Way, Oxford, UK; ^3^​ Health Protection Research Unit in Healthcare Associated Infections and Antimicrobial Resistance, University of Oxford, Oxford, UK

**Keywords:** antimicrobial resistance, compensatory mutations, fitness cost, tuberculosis

## Abstract

Mutations in *Mycobacterium tuberculosis* associated with resistance to antibiotics often come with a fitness cost for the bacteria. Resistance to the first-line drug rifampicin leads to lower competitive fitness of *M. tuberculosis* populations when compared to susceptible populations. This fitness cost, introduced by resistance mutations in the RNA polymerase, can be alleviated by compensatory mutations (CMs) in other regions of the affected protein. CMs are of particular interest clinically since they could lock in resistance mutations, encouraging the spread of resistant strains worldwide. Here, we report the statistical inference of a comprehensive set of CMs in the RNA polymerase of *M. tuberculosis*, using over 70 000 *M*. *tuberculosis* genomes that were collated as part of the CRyPTIC project. The unprecedented size of this data set gave the statistical tests more power to investigate the association of putative CMs with resistance-conferring mutations. Overall, we propose 51 high-confidence CMs by means of statistical association testing and suggest hypotheses for how they exert their compensatory mechanism by mapping them onto the protein structure. In addition, we were able to show an association of CMs with higher *in vitro* growth densities, and hence presumably with higher fitness, in resistant samples in the more virulent *M. tuberculosis* lineage 2. Our results suggest the association of CM presence with significantly higher *in vitro* growth than for wild-type samples, although this association is confounded with lineage and sub-lineage affiliation. Our findings emphasize the integral role of CMs and lineage affiliation in resistance spread and increases the urgency of antibiotic stewardship, which implies accurate, cheap and widely accessible diagnostics for *M. tuberculosis* infections to not only improve patient outcomes but also prevent the spread of resistant strains.

## Data Summary

All genetic sequences used have been previously published and deposited in the ENA, as described in the source paper [1]. Data tables focusing on our genes of interest were derived from the larger CRyPTIC data tables that are publicly available via an FTP site [1]. These can be found, along with Python code, in form of a jupyter-notebook, allowing one to recreate the analysis and thence figures in this paper in a GitHub repository [2].

**Impact Statement**

Mutations that confer resistance to an antimicrobial often confer a fitness disadvantage that can be alleviated by compensatory mutations elsewhere in the protein complex. We have produced a comprehensive list of putative compensatory mutations in the RNA polymerase of *M. tuberculosis* that recover the fitness cost introduced by mutations conferring resistance to rifampicin, a key drug in the standard treatment regimen for tuberculosis. This is the most extensive study to date, making use of over 70 000 clinical genomes, and we were also able to use growth data on a subset of 13 990 samples to demonstrate how the size of the effect varies depending on the mutations and the lineage of the sample.

## Introduction

The rise of multidrug-resistant (MDR) bacteria is one of the grand challenges we are facing as a global society. A study in 2019 found 7.7 million deaths per year associated with bacterial infections [3]. This ongoing crisis is mainly caused by antimicrobial resistance arising within short time frames and spreading rapidly throughout bacterial populations, reinforced by directional selection through inappropriate administration of antibiotics [4, 5]. New antibiotic drugs are being developed but the pace is slow and the rate at which resistance to new drugs develops is higher [6]. With the increasing prevalence of antibiotic resistance and the decreasing discovery rate of potent new drugs, we are heading towards a human-fabricated global health crisis [4, 7].

*Mycobacterium tuberculosis* is the aetiological agent of tuberculosis (TB) and prone to developing resistance to major antibiotics [8]. Hence, although the first antibiotic for treatment of TB was identified as early as 1948 [9], the disease remains responsible for the death of ~1.6 million people per year. This is in part due to funding for prevention, diagnosis and treatment of TB continuously falling short of the amount required to implement national strategic plans. In 2021, this shortfall reportedly amounted to USD $1.6 billion [10]. In addition, the proportion of people infected with *M. tuberculosis* strains resistant to the major first-line antibiotic drugs is rising [10–12]. Understanding how resistance mutations emerge, how they become fixed and how they spread is hence of high importance.

**Fig. 1.**
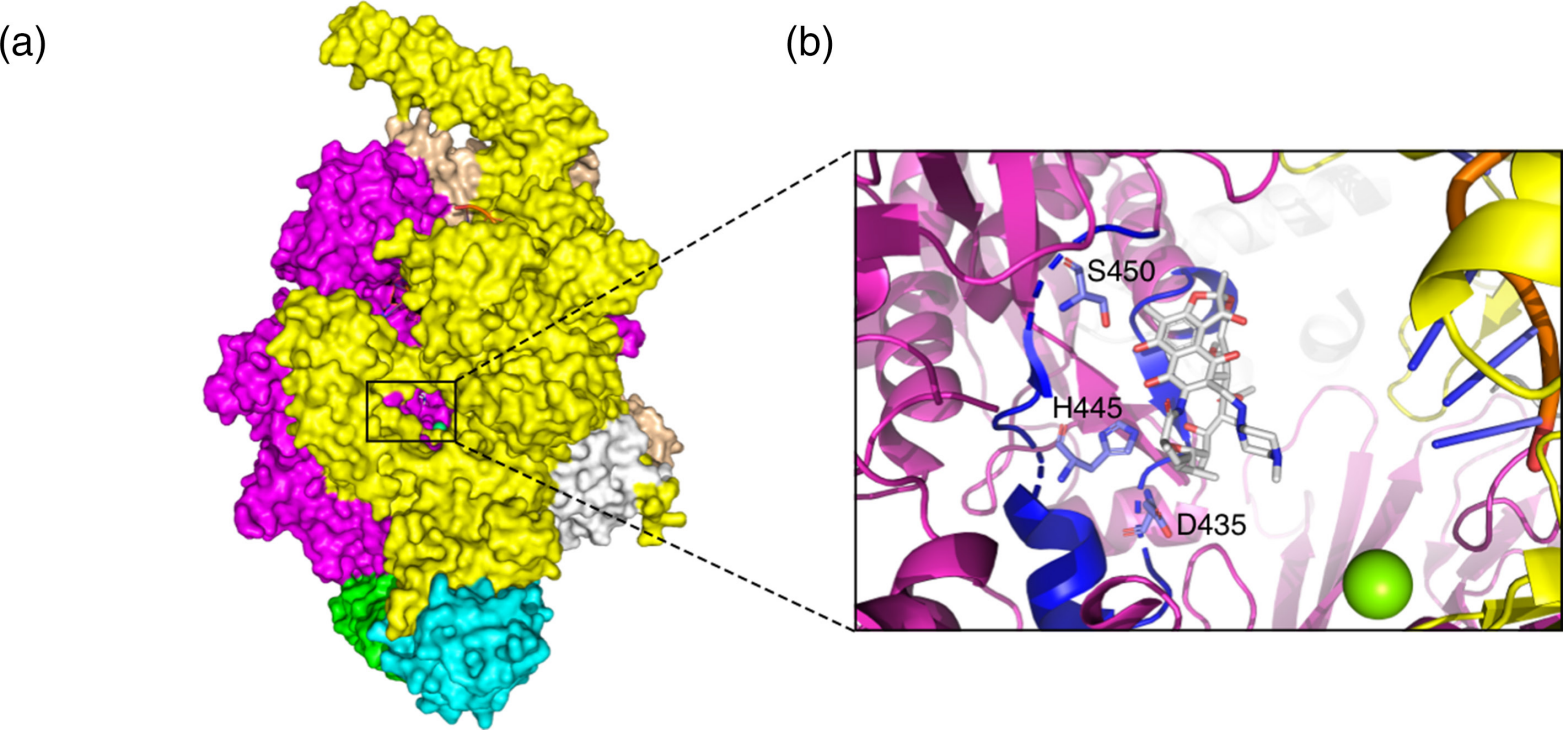
The drug rifampicin (RIF) interrupts RNA synthesis by binding to the *β* subunit of the RNA polymerase (RNAP) [14]. (**a**) Overview of the entire RNAP. The *β* subunit of the RNAP is shown in magenta, the β′ subunit in yellow, the two α subunits in light blue and green, the ω subunit in white and the σ factor in light orange. The active site is framed in black. It can be seen through the secondary channel, with the active site magnesium depicted in green and the drug RIF in white. (**b**) Close-up of the active site. The DNA strand used as a template for transcription is shown on the right in orange and dark blue. The β subunit of the RNAP is shown in magenta, with the rifampicin resistance-determining region (RRDR) highlighted in dark blue. The protruding amino acids D435, H445 and S450 are reported to form hydrogen bonds or van der Waals interactions with the drug RIF [14]. Due to the proximity of the RRDR to the RNAP active centre, the binding of RIF causes a disruption of the RNA synthesis through steric clash.

One of the four first-line antibiotics for treating TB is the drug rifampicin (RIF), which binds to the RNA polymerase (RNAP). The RNAP holoenzyme in *M. tuberculosis* is composed of five subunits (α) and the σ factor (Fig. 1a) [13]. RIF binds close to the active site in the β subunit (*rpoB* gene). In susceptible bacteria, amino acids within the so-called rifampicin resistance-determining region (RRDR) form hydrogen bonds with RIF (Fig. 1b). The bound RIF sterically obstructs the elongation of newly synthesized RNA, thereby stalling protein production in the bacteria [14]. Since *M. tuberculosis* exhibits very little evidence of horizontal gene transfer [15], resistance to this drug mostly arises through chromosomal mutations within or close to the RRDR that prevent RIF from binding [16–18]. These resistance-conferring mutations introduce different amino acid side chains close to the active site of the RNAP, hence it is not surprising that they also introduce a fitness cost [19, 20]. This fitness cost in RIF-resistant bacteria manifests in decreased performance in competition assays with susceptible bacteria [20]. While the molecular basis of the fitness deficit is not entirely understood, it is suspected to be related to decreased stability of the RNAP open promoter complexes [21], as well as steric hindrance of RNA exit by the mutated amino acids [22]. The low fitness phenotype has been observed to be partially or completely rescued by the presence of so-called compensatory mutations (CMs) [23–25]. These CMs emerge in various subunits of the RNAP [24] and have been shown to partially restore RNAP activity in *Mycobacterium smegmatis* [25]. The existence of CMs gives a plausible explanation for the persistence of resistance mutations long after antibiotic treatment is stopped, when the fitness cost should lead to reversion to the wild-type phenotype.

Due to their potential to lead to fixation of resistance mutations [22] and the attendant epidemiological consequences, CMs have been extensively investigated. Many candidates have been identified in previous studies [26–32], but the number of samples available to these studies is often small. Furthermore, the effects on growth and/or polymerase activity have only been experimentally confirmed for a small subset of putative CMs [25, 31]. To further our understanding of resistance spread and persistence, it would be useful to construct a more comprehensive list of CMs and, if possible, to dissect their direct influence on the fitness of *M. tuberculosis*.

The identification of CMs is not trivial and depends on how they are defined. In some publications, due to the low number of available samples, CMs are simply assumed to be all mutations in the RNAP that co-occur with resistance mutations [28]. Higher numbers of samples allowed CMs to be defined as mutations that *exclusively* occur with resistance mutations [26, 30, 32]. With the data set of 77 860 *M*. *tuberculosis* sample genomes we have at hand, where sequencing errors and sample mislabelling are likely to lead to a considerable amount of false positives, it is necessary to employ statistical association testing.

In this paper, we identify a comprehensive set of 51 putative CMs based on a Genetics data set comprising 77 860 *M*. *tuberculosis* sample genomes collated by the international CRyPTIC project [1]. The large size of the data set ensures the statistical analysis is ~10–100 times more powerful than those of previous studies [26–32]. Another advantage of our investigation is that we have *in vitro* growth data available for a subset of 13 990 samples derived from photographs of 96-well plates after 2 weeks’ incubation – we shall call this the Growth data set [33]. This allows us to correlate the observed growth phenotypes with the respective genotype, i.e. the presence of resistance-conferring and compensatory mutations.

## Methods

### Reproducibility statement

The majority of figures and tables in this report can be reproduced using a GitHub repository and attendant jupyter notebook available online [2].

### Data set sources

The Genetics data set comprised 77 860 whole-genome-sequenced patient-derived samples of *M. tuberculosis* collected and collated by the CRyPTIC project [1, 34]. Mutations with respect to version 3 of the H37Rv reference genome were aggregated and are available in a series of data tables, along with other data, such as the predicted resistances to various antibiotics, ENA accession numbers, lineage association and the amount of growth in the 96-well plates [35].

A subset of 13 990 samples (the Growth data set) were inoculated onto 96-well broth microdilution plates, incubated for 2 weeks and then a photograph was taken by the CRyPTIC project using a Thermo Fisher Sensititre Vizion Digital MIC Viewing System [36, 37]. Each photograph was subsequently analysed and the mean bacterial growth, as measured by AMyGDA, of the two positive control wells was recorded as described elsewhere [33]. We shall refer to this as the Growth data set. The bacterial growth is the percentage of dark pixels in a circle centred on the well but with half the radius (to avoid edge artefacts and shadows) and therefore can reach 100 %.

### Pairwise statistical association testing

Using the Genetics data set we compiled a list of putative compensatory mutations (CMs) by performing statistical association testing for all mutations in the RNA polymerase (RNAP) genes that were observed to co-occur with any mutations listed as conferring resistance to rifampicin (RIF) in the catalogue [35]. We excluded any samples where sequencing was inconclusive. The statistical test used was Fisher’s exact test, which has a high computational cost that increases with data set size. To minimize the effect of this, we used a Python 3 package with an optimized implementation of Fisher’s test. This is faster than the standard implementation (fishers-exact-test) and enabled us to run Fisher’s exact test for every pair of resistance and co-occurring mutation within 2.5 h on a modern workstation.

### Evaluating and filtering results from Fisher’s exact test

Despite being generally accepted as a conservative approach to correct for multiple testing, naively applying a Bonferroni-corrected *P*-value of 0.01 % (Fig. S2) to the results of the pairwise Fisher’s exact test leads to hundreds of hits and a TPR of 100 %, most likely due to the inflating effect of linkage disequilibrium (LD) on *P*-values as mentioned in the Results section. We decided to only include hits with a *P*-value above the 98 % percentile, since this still recovers 84.6 % of the high-confidence CMs and gives us a reasonable number of hits that we can process using our downstream filters. The entire list without *P*-value cut-off is available online [2]. To make sure we filter out any phylogenetic mutations, we removed any synonymous mutations and tested the remaining for homoplasy. Synonymous mutations tend to be phylogenetic markers since they do not change the protein structure and hence should not be subject to any selectional pressure. Homoplasy is an indicator of selective pressure that causes similar mutations to arise in genetically and evolutionary distinct populations. Our resulting preliminary list of CMs, including their respective associated resistance mutation, is shown in Table S2.

### Constructing binary homoplasy index

The CRyPTIC consortium constructed a phylogenetic tree [1, 38] from 15 211 isolates, which is the number of samples collected that have both genetic data and minimum inhibitory concentrations available. They constructed the tree based on the pairwise genetic distance matrix, where a neighbourhood-joining tree was visualized and annotated in R using the ggtree library and the lineages were assigned using Mykrobe. We mapped our putative hits on the tree using the publicly available software iTOL [39]. Any samples originating from organisms other than *M. tuberculosis* (e.g. *Mycobacterium caprae* and *Mycobacterium bovis*) and lineages other than 1–4 were removed. We also removed any samples where sequencing was inconclusive, since these were initially excluded from our statistical association testing. We inferred homoplasy if a mutation appeared in at least three genetically distant samples without direct common ancestors in the tree. This number was chosen to partly account for the possibility of false positives due to sequencing errors or hemiplasies. It should be noted that if a mutation was not classified as homoplastic, this does not exclude the possibility of this mutation being a CM.

### Plotting of growth data

The average growth, as measured by AMyGDA [33], of the two positive control wells for each sample was obtained from the CRyPTIC data set. To quantify the differences between growth of samples that are pan-susceptible, resistant or resistant with CMs, the pairwise Mann–Whitney *P*-value for the two growth distributions was calculated. In addition, the medians and confidence intervals of the medians were approximated using a bootstrapping approach and included in notched box-plot figures. This is a robust method considering the non-normal distribution of the growth data. For significance, we always referred to the Mann–Whitney *P*-values.

### Mapping of putative compensatory mutations on RNA polymerase structure

High-confidence homoplastic hits were mapped in PyMOL onto the crystal structure of the *M. tuberculosis* RNAP in complex with RIF [14] to identify clusters and elucidate potential compensatory mechanisms.

## Results

### Rifampicin-resistant *M. tuberculosis* samples show lower *in vitro* growth densities than pan-susceptible samples

First, we analysed the effect of resistance-conferring mutations on the *in vitro* growth of *M. tuberculosis* bacteria. Based on our Growth data set of 13 990 whole-genome-sequenced patient-derived *M. tuberculosis* samples with associated 96-well growth data, we defined 2 sets of samples: one resistant to rifampicin (RIF) and another that is susceptible. Resistance was defined as the presence of any mutation associated with resistance to RIF according to a published catalogue [35], whilst a sample was assumed to be susceptible if the catalogue classified it as pan-susceptible (susceptible to all four first-line tuberculosis drugs – RIF, isoniazid, ethambutol and pyrazinamide). We excluded samples containing any other non-synonymous mutations in the RNA polymerase (RNAP), since they could have a secondary effect on growth. That being said, we cannot exclude an effect due to mutations in other genes. We shall further assume that synonymous mutations in the RNAP have no effect on the growth phenotype, and hence need not be excluded. The resulting sample sizes can be seen in Table 1.

**Table 1.** Median growth of samples with and without indicated resistance mutations, where growth is represented by the percentage of a well containing bacterial growth as measured by the CRyPTIC project. The confidence interval (CI) for the median is calculated using bootstrapping where ‘CI low’ indicates the lower threshold and ‘CI high’ the upper threshold. The Mann–Whitney *P*-value is calculated in reference to pan-susceptible sample growth and *n* indicates the sample size

Mutation	Median growth [%]	CI low	CI high	*P*-value	*n*
Pan-susceptible	22.1	21.6	22.7		5283
Any resistance	17.6	16.6	18.5	2.95e-12	795
Specific resistance mutation:					
*rpoB* S450L	16.7	15.1	18.9	4.37e-03	196
*rpoB* H445Y	15.5	12.0	19.2	1.26e-02	42
*rpoB* D435V	16.4	15.5	17.2	3.92e-14	325

**Fig. 2.**
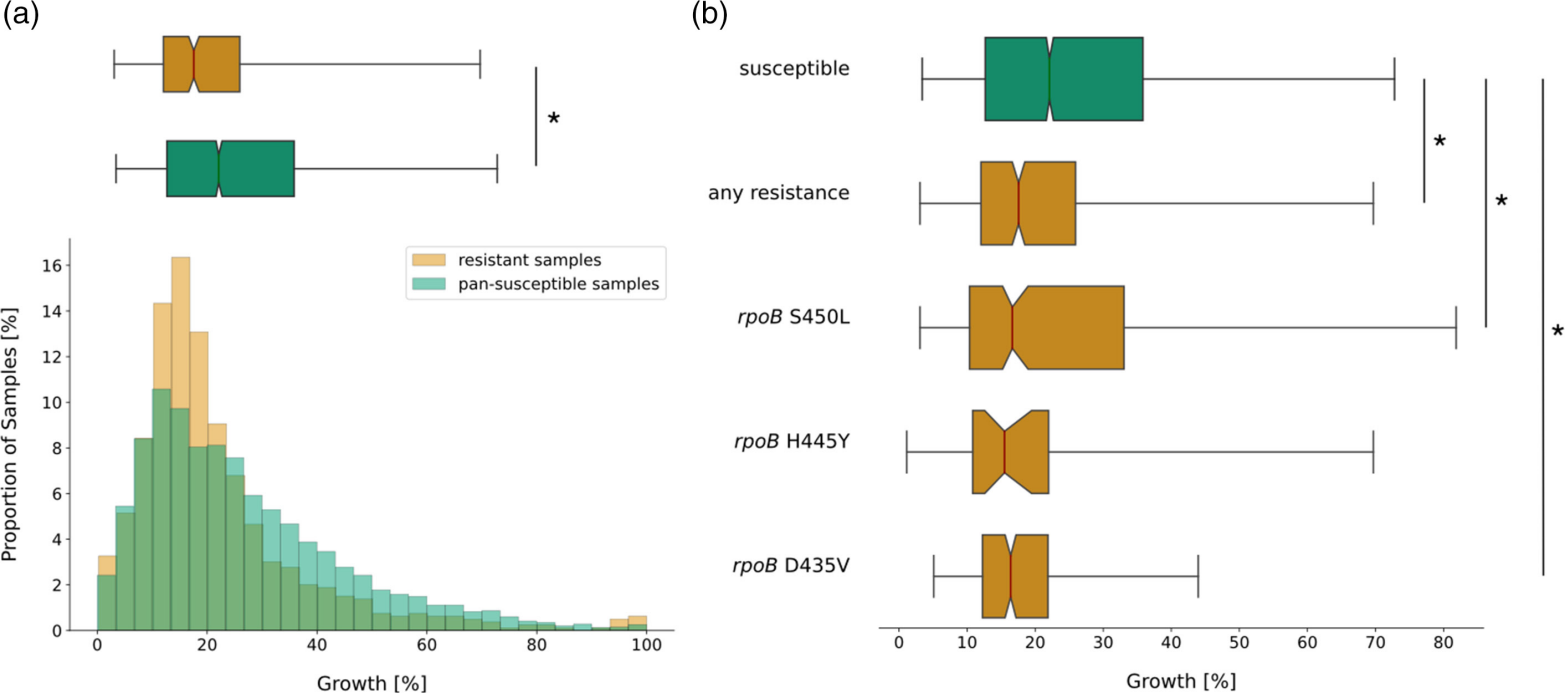
Presence of rifampicin (RIF) resistance-conferring mutations in the RNA polymerase (RNAP) of *M. tuberculosis* is associated with lower median growth compared to pan-susceptible samples. (**a**) Distributions of growth in percentage of covered well area as measured in the CRyPTIC project [33] were plotted as a histogram against the proportion of samples that display this amount of growth (bottom) and as a notched box plot reflecting the distribution quantiles (top). Samples with RIF resistance mutations but no other potentially interfering mutations are plotted in red and samples classified as pan-susceptible are plotted in green. For the box plot, half of the data lie within the area of the box and 95 % in the area covered by the whiskers. Outliers (5 % of the data) were removed to achieve a cleaner representation. Indented areas close to the medians indicate their respective confidence intervals, while the asterisk (*) indicates a significant Bonferroni-corrected Mann–Whitney *P*-value (*P*<0.05/10 %). The respective medians, confidence intervals and the Mann–Whitney *P*-value are listed in Table 1. (**b**) Plot structure equivalent to the box plot in (a), but the red bars represent subsets of RIF-resistant samples that exhibit only the resistance mutation indicated to their left and no other potentially interfering mutations. The medians, their confidence intervals (CIs) and Mann–Whitney *P*-values of the distributions are listed in Table 1. For a histogram representation of the data please refer to Fig. S1, available in the online version of this article.

There is a significant difference between the growth distributions of our sets (Fig. 2a), as confirmed by a significant Mann–Whitney *P*-value of 2.95e-12 (Table 1). The median growth density in resistant samples was significantly lower than in pan-susceptible samples (4.5 % absolute growth difference), hence we conclude that we see higher growth in non-resistant bacteria.

This agrees with work by Gagneux *et al.* who reported that RIF resistance introduces a fitness cost in *M. tuberculosis*, which they show by *in vitro* competitive fitness experiments [20]. Their study indicates that the magnitude of the fitness cost in *M. tuberculosis* depends on the mutation, with *rpoB* S450L, the most prevalent RIF resistance mutation observed clinically, being associated with the lowest fitness cost [20]. We had sufficient samples to investigate growth for the three most common resistance mutations in the data set: S450L, H445Y and D435V. Consistent with Gagneux *et al.*, the median growth of *rpoB* S450L mutants was the highest among the three resistance mutations (0.3 and 1.2 % absolute growth difference, respectively), while still growing to significantly lower densities than pan-susceptible samples (5.4 % absolute growth difference, *P*=4.37e-03, Fig. 2b, Table 1). However, the difference in median growth between the three resistance mutations is non-significant (Table S1), presumably due to the number of eligible samples being too low.

### The causal relationship of compensatory mutations with resistance allows the identification of compensatory mutations through homoplasy

Given that samples with resistance-conferring mutations grow less well than the wild-type, one expects to detect additional mutations in the population that have subsequently evolved in resistant samples to restore, at least partially, this apparent drop in fitness. To identify these mutations we performed a Fisher’s exact test for each pair of resistance and co-occurring RNAP mutation in the Genetics data set, to determine if the latter is significantly associated with resistance (Methods).

For a largely clonal species like *M. tuberculosis*, linkage disequilibrium (LD) will artificially inflate the *P*-values in any SNP–phenotype association test, as can be seen in most genome-wide association studies of microbial species [40]. LD can hence mask true causal mutation–phenotype relationships, like that between RIF resistance and CM presence. Furthermore, classic stratification corrections alone will not be sufficient to identify causal variants in species with strong LD and the use of homoplasy as a criterion has been proposed instead [41]. We hence decided to apply a conservative and arbitrary *P*-value cut-off to counter the *P*-value inflation, and additionally excluded any hits that are not homoplastic. To assess the performance of our approach, we compiled a list of CMs from the literature. Since all these CMs have either been confirmed experimentally or have been identified by at least three published studies, we shall assume that they are high-confidence CMs (Table S2). We shall use this compiled reference list to assess our arbitrary choice of *P*-value cut-off by considering the true positive rate (TPR, Fig. S2).

We were able to recover 84.6 % (15/17, Table S2) of these putative high-confidence CMs using our arbitrary *P*-value cut-off set at the 98 % interval, equivalent to a *P*-value cut-off at *P*=1e-25.9. This results in a preliminary list of mutations that are significantly associated with RIF resistance. Synonymous mutations are unlikely to have a strong effect on the growth phenotype, as they do not change the structure of the final protein and thus they were removed from the list. The resulting list contained 78 putative CMs (Table S3). Of note ~70 % of hits were significantly associated with the resistance mutation S450L, which indicates a general strong association of this particular resistance mutation with the presence of CMs.

**Fig. 3.**
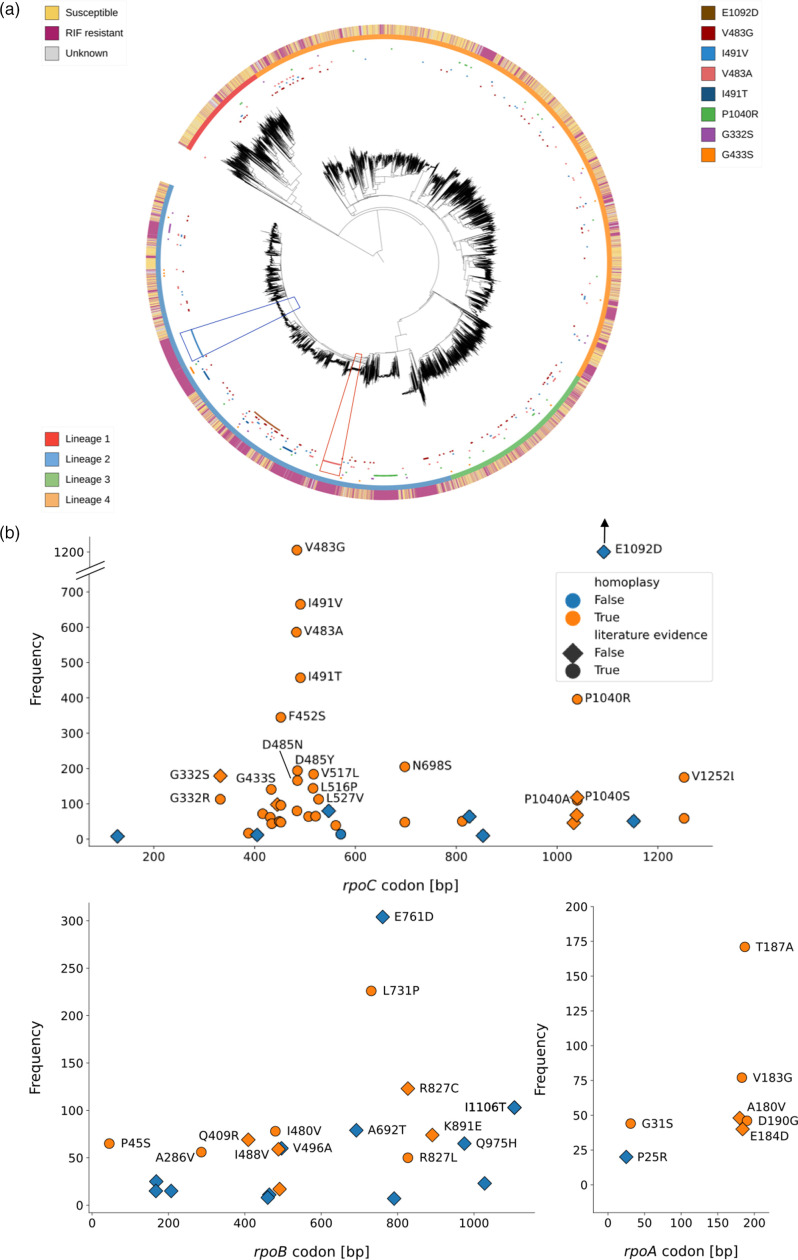
Putative CMs are distributed widely across the phylogenetic tree and the RNA polymerase genes (**a**) Phylogenetic tree assembled from single-nuceotide polymorphism (SNP)-distances of about 15,000 samples. The resistance level of the samples is indicated on the outermost ring, while the second ring indicates the lineage. The most common CMs (all on *rpoC*) were mapped on the innermost ring, with the trapezoids indicating clades within lineage 2 that show a cluster of a specific CM (I491V, blue trapezoid; V483A, red trapezoid). (**b**) The putative CMs were mapped according to their position in the respective gene and their frequency of co-occurrence with resistance. There were five hits on the σ factor that are not shown, as well as one hit on the *rpoZ* gene (Table S3). All of these do not show homoplasy. E1092D is outside of the plotting range due to its high frequency (1989 observations).

We mapped these 78 putative CMs onto a phylogenetic tree of the *M. tuberculosis* samples (Methods). The majority of the most frequent putative CMs in our data set indeed show homoplasy (Fig. 3a). But the putative CM *rpoC* E1092D for example exclusively occurred in a single clade of lineage 2 and is thus highly likely to be a phylogenetic marker rather than a CM. We observed clustering in clades of lineage 2 for other CMs (Fig. 3a: P1040R, I491V: blue trapezoid and V483A: red trapezoid), but in contrast to E1092D, these CMs also occurred in other, genetically distant parts of the tree. This makes convergent evolution of compensation more likely than homology.

After filtering out hits that do not exhibit homoplasy, we arrived at a final list of 51 putative CMs. Out of these 51 hits, 12 have to our knowledge not been previously described (Fig. 3b, Table S3). All of these hits would be valid starting points for further investigations of compensatory effects and mechanisms. In this final list, the association of CMs with the resistance mutation S450L in particular is even more apparent, with this resistance mutation accounting for 92 % of CM associations.

### Rifampicin-resistant *M. tuberculosis* samples show higher *in vitro* growth densities in presence of compensatory mutations in lineage 2

**Fig. 4.**
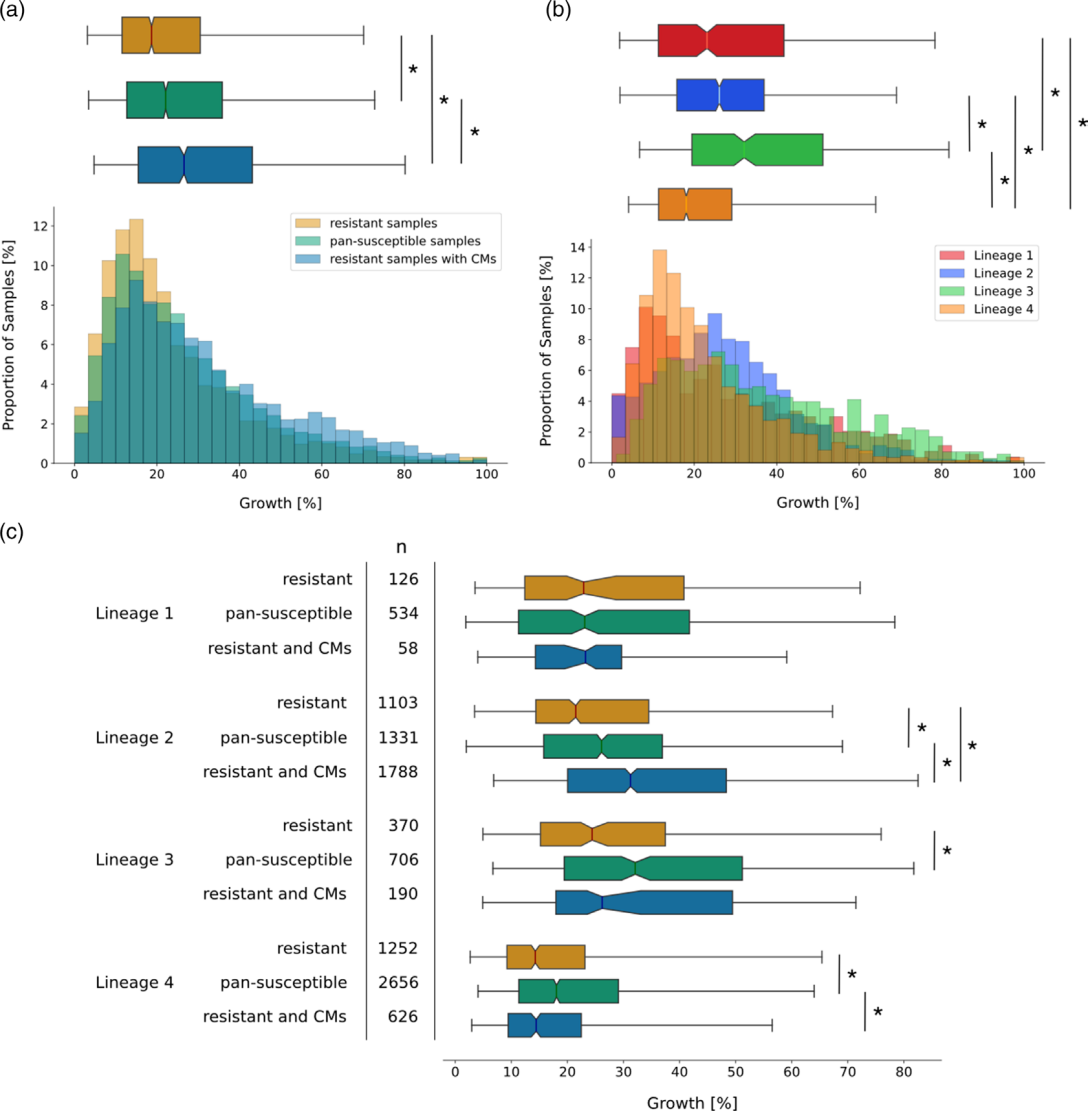
Presence of compensatory mutations (CMs) in samples with rifampicin (RIF) resistance-conferring mutations in the RNA polymerase of *M. tuberculosis* is associated with higher growth densities in some lineages. (**a**) Distributions of growth in percentage of covered well-area as measured in the CRyPTIC project [33] were plotted as a histogram against the proportion of samples that display this amount of growth (bottom) and as a notched box plot reflecting the distribution quantiles (top). Samples with RIF resistance mutations but no putative CMs are plotted in red and samples classified as pan-susceptible are plotted in green. Samples that have RIF resistance mutations and at least one CM are shown in blue. For the box plot, half of the data lie within the area of the box and 95 % in the area covered by the whiskers. Outliers (5 % of the data) were removed to achieve a cleaner representation. Indented areas close to the medians indicate their respective confidence interval, while the asterisk (*) indicates a significant Bonferroni-corrected Mann–Whitney *P*-value (*P*<0.05/3 %). The respective medians, confidence intervals and the Mann–Whitney *P*-values are listed in Table S4. (**b**) Plot structure equivalent to the box plot in (a), but the bars represent pan-susceptible samples from different lineages, plotted in the respective colours indicated in the legend. The asterisk (*) indicates a significant Bonferroni-corrected Mann–Whitney *P*-value (*P*<0.05/6 %). The respective medians, confidence intervals and the Mann–Whitney *P*-value are listed in Table S5. (**c**) Plot structure equivalent to the box plot in (a), but the box plots represent subsets of samples that belong to the lineage displayed on the left. The sample size is shown in the column marked with *n*. For a histogram representation of the same data refer to Fig. S4. The asterisk (*) indicates a significant Bonferroni-corrected Mann–Whitney *P*-value (*P*<0.05/3 %). The respective medians, confidence intervals and the Mann–Whitney *P*-values are listed in Table S6.

If we have correctly identified CMs, growth densities in the resistant samples with CMs should be higher than in resistant samples without CMs in our Growth data set. To test this, we compared the growth distributions of pan-susceptible samples, RIF-resistant samples that contain at least one homoplastic CM from our complete list (Table S3), and resistant samples without any CMs. Resistant samples with CMs grew to significantly higher densities (7.9 % absolute growth difference, *P*=3.92e-57) than resistant samples without CMs (Fig. 4a, Table S4). Surprisingly, the median growth of resistant samples with CMs also significantly out-performed the growth of pan-susceptible samples (4.5 % absolute growth difference, *P*=6.25e-26, Fig. 4a, Table S4). The association of CMs with such high growth levels could mean that CMs increase fitness above wild-type levels, or hint at confounding factors.

One confounding factor could be rooted in the differences in virulence between *M. tuberculosis* lineages. Lineage 2 is thought to be associated with higher transmission rates than other lineages [42], which could be reflected in higher *in vitro* growth densities. Indeed, considering only pan-susceptible samples, lineage 2 showed significantly higher growth than lineage 4 (8 % absolute growth difference, *P*=7.99e-33) and higher growth than lineage 1 (3 % absolute growth difference, *P*=7.17e-02), whilst still being out-performed by lineage 3 (6 % absolute growth difference, *P*=8.36e-16, Fig. 4b, Table S5). In addition, 60 % of resistant samples in lineage 2 contained at least one CM from our final list, compared to 34 % in other lineages. Higher growth densities of lineage 2 combined with CM accumulation could lead to inflated growth phenotype association with CMs.

We hence need to examine lineage contribution to the enhanced growth phenotype in CM samples. In lineage 2 we still found a significantly higher growth density for samples with CMs than for pan-susceptible samples (5.3 % absolute growth difference, *P*=3.20e-24, Fig. 4c). For all other lineages, the absolute growth difference between resistant samples with CMs and pan-susceptible samples was either insignificant, or pan-susceptible samples grew to higher densities (Fig. 4c, Table S6). In lineage 3 we observed a slightly higher growth density of resistant samples with CMs when compared to resistant samples without CMs (1.9 % absolute growth difference, *P*=2.07e-02), but this was insignificant after applying Bonferroni correction, probably due to low sample size. This indicates that while CMs partially restore fitness, the extreme growth phenotypes are not caused by CMs alone. There might be other growth-associated mutations in lineage 2, acting as confounding factors. We do not observe an effect on growth in lineages 1 and 4. In lineage 1, the sample size might be too low to detect an effect on growth, since we could not even observe the initial fitness cost in resistant samples without CMs (Fig. 4c, Table S6). Lineage 4, on the other hand, showed very low overall growth (Fig. 4b), which makes it difficult to detect the positive effect of CMs due to the relatively small initial effect of resistance mutations on growth (Fig. 4c).

### The effect of compensatory mutations on *in vitro* growth is confounded with lineage and clade affiliation

Lineage 2 is the only lineage that showed significantly higher growth in resistant samples with CMs than in pan-susceptible samples while also accumulating CMs more than any other lineage in our Growth data set. While this might indicate that CMs play an important role in this lineage, we suspected that there might be other mutations co-occurring with CMs that enhanced growth densities to the reported high levels.

**Fig. 5.**
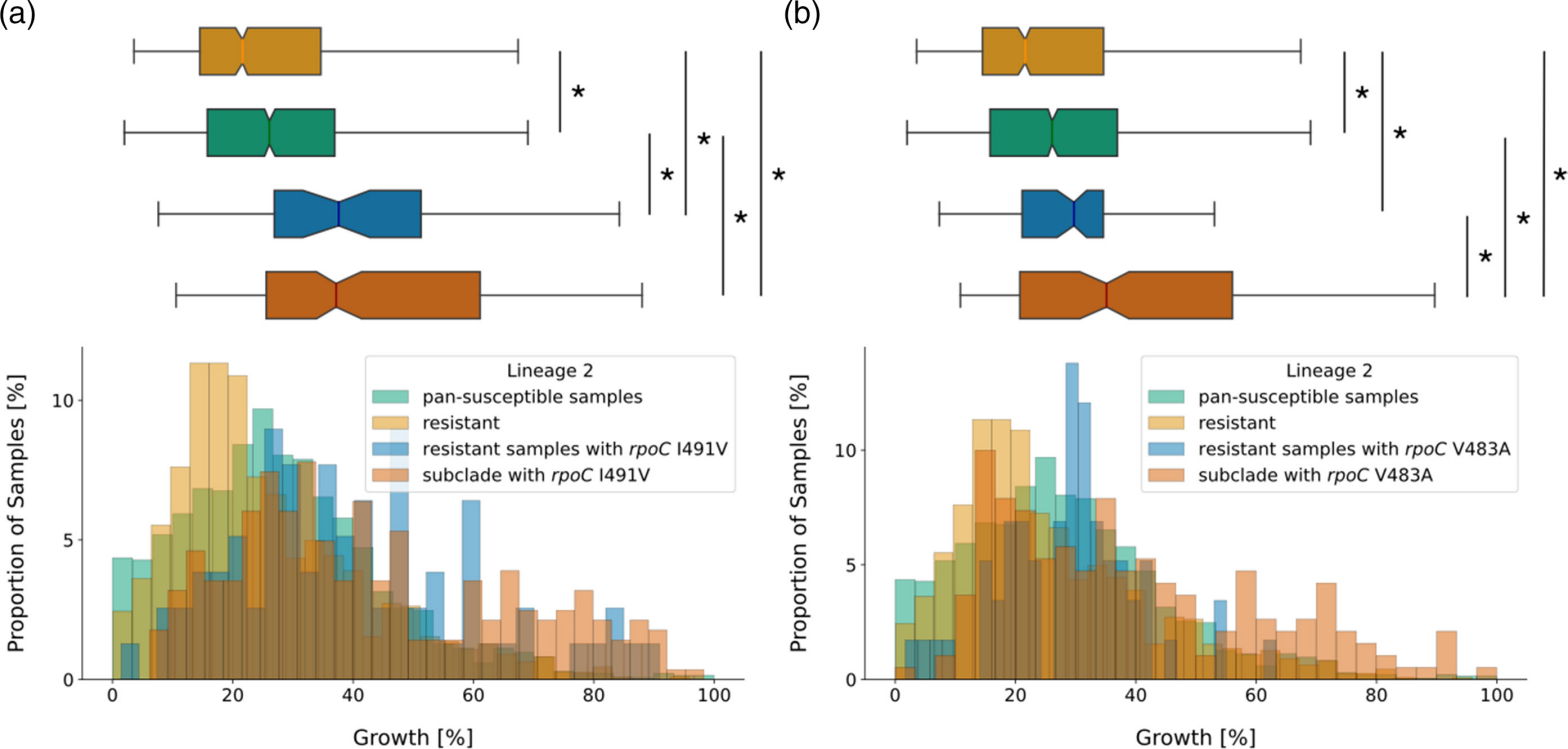
*M. tuberculosis* clades with clusters of compensatory mutations (CMs) explain some of the high growth densities associated with CMs in lineage 2. (**a**) Growth distributions (percentage of covered well area) in lineage 2 were plotted as a histogram against the proportion of samples that display this amount of growth (bottom) and as a notched box plot reflecting the distribution quantiles (top). Lineage 2 samples were classified as pan-susceptible (green), RIF resistant (red), resistant and showing the CM I491V outside of the CM cluster clade (blue) and as part of the lineage 2 clade, where all samples show the CM I491V (dark red). For the box plot, half of the data lie within the area of the box and 95 % in the area covered by the whiskers. Outliers (5 % of the data) were removed to achieve a cleaner representation. Indented areas close to the medians indicate their respective confidence interval, while the asterisk (*) indicates a significant Bonferroni-corrected Mann–Whitney *P*-value (*P*<0.05/6 %). The respective medians, confidence intervals and the Mann–Whitney *P*-values are listed in Table 2. (**b**) Plot structure equivalent to the box plot in (a), but the CM in question is V483A. Distribution medians and their confidence intervals are shown in Table 2.

We hence wanted to investigate whether the high growth densities observed in samples with CMs within lineage 2 reflect the growth advantage of a few high-fitness clades. We aimed to dissect the clade influence for two representative CM clusters with sufficient available samples (Fig. 3a), specifically setting a cut-off at *n*=50 for each sample group to obtain meaningful, significant statistics. The first cluster showed accumulation of the CM *rpoC* I491V (blue trapezoid in Fig. 3a). The average SNP distance of 23 within the cluster indicated that this cluster is not the result of recent transmission according to UK Health Security Agency (UKHSA) guidelines [43], although the maximum SNP distance of 44 highlights that samples are nonetheless very closely related. Most likely the cluster is indeed an established clade within lineage 2 and not an oversampled local outbreak. We saw that the clade with the *rpoC* I491V cluster showed significantly higher growth than pan-susceptible samples (11.1 % absolute growth difference, *P*=6.52e-24), but the effect outside of this clade was just as strong (11.5 % absolute growth difference, *P*=1.12e-07, Fig. 5a, Table 2). The second cluster showed accumulation of the CM *rpoC* V483A (red trapezoid in Fig. 3a). The average SNP distance was higher than in the other cluster, with ~31 pairwise SNP differences between samples in the cluster and a maximum SNP distance of 149. We saw that median growth in the clade with the *rpoC* V483A cluster was significantly higher than in samples with this CM outside of the clade (5.4 % absolute growth difference, *P*=6.79e-03). But median growth of the latter was still significantly higher than the median growth of resistant samples without any CMs (8.1 % absolute growth difference, *P*=4.24e-03, Fig. 5b, Table 2), which suggests that a small positive effect of the CM on growth is conserved after removing the confounding influence of the high-fitness clade. However, the increase of growth over pan-susceptible growth levels is not significant outside of the high-fitness clade for the CM V483A (3.6 % absolute growth difference, *P*=0.263, Fig. 5b, Table 2), which implies that the clade association is a confounding factor in this case.

**Table 2.** Median growth of samples from lineage 2 with different compensatory mutations, compared to growth of pan-susceptible samples, growth of resistant samples without CMs and growth of a resistant clade that shows a cluster of the respective CM. Growth is represented by the percentage of a well containing bacterial growth as measured by the CRyPTIC project. The confidence interval (CI) for the median is calculated using bootstrapping where 'CI low' indicates the lower threshold and 'CI high' the upper threshold. *P*-values are given with respect to resistant sample growth (*P*-value (r)), pan-susceptible sample growth (*P*-value (s)) and sample growth of resistant samples with CMs outside of the cluster subclade (*P*-value (rCM)). *n* indicates the sample size

Sample type	Median growth [%]	CI low	CI high	*P*-value (r)	*P-*value (s)	*P*-value (rCM)	*n*
Pan-susceptible	26.08	25.21	26.99				1331
Resistant and no CMs	21.6	20.5	22.4		2.46e-05		1103
CM *rpoC* I491V:							
Resistant	37.6	31.5	42.3	9.57e-11	1.12e-07		78
Resistant and subclade	37.2	33.9	41.4	2.14e-33	6.52e-24	0.499	282
CM *rpoC* V483A:							
Resistant	29.7	26.9	31.7	4.24e-03	0.263		58
Resistant and subclade	35.1	30.7	38.9	9.45e-17	9.89e-11	6.79e-03	190

The association of CM clusters with specific high-fitness clades within lineage 2 hence can be a confounding factor in our growth data analysis and might explain a part of the observed increase of growth densities to levels higher than the wild-type growth densities. Further work is needed to completely disentangle the influence of these high-fitness clades on the growth phenotype. However, the small growth advantage of the resistant sample with CMs over the resistant sample without CMs appears to be conserved even when these clades are removed from the analysis.

### Most compensatory mutations are found at interfaces between the RNA polymerase subunits

**Fig. 6.**
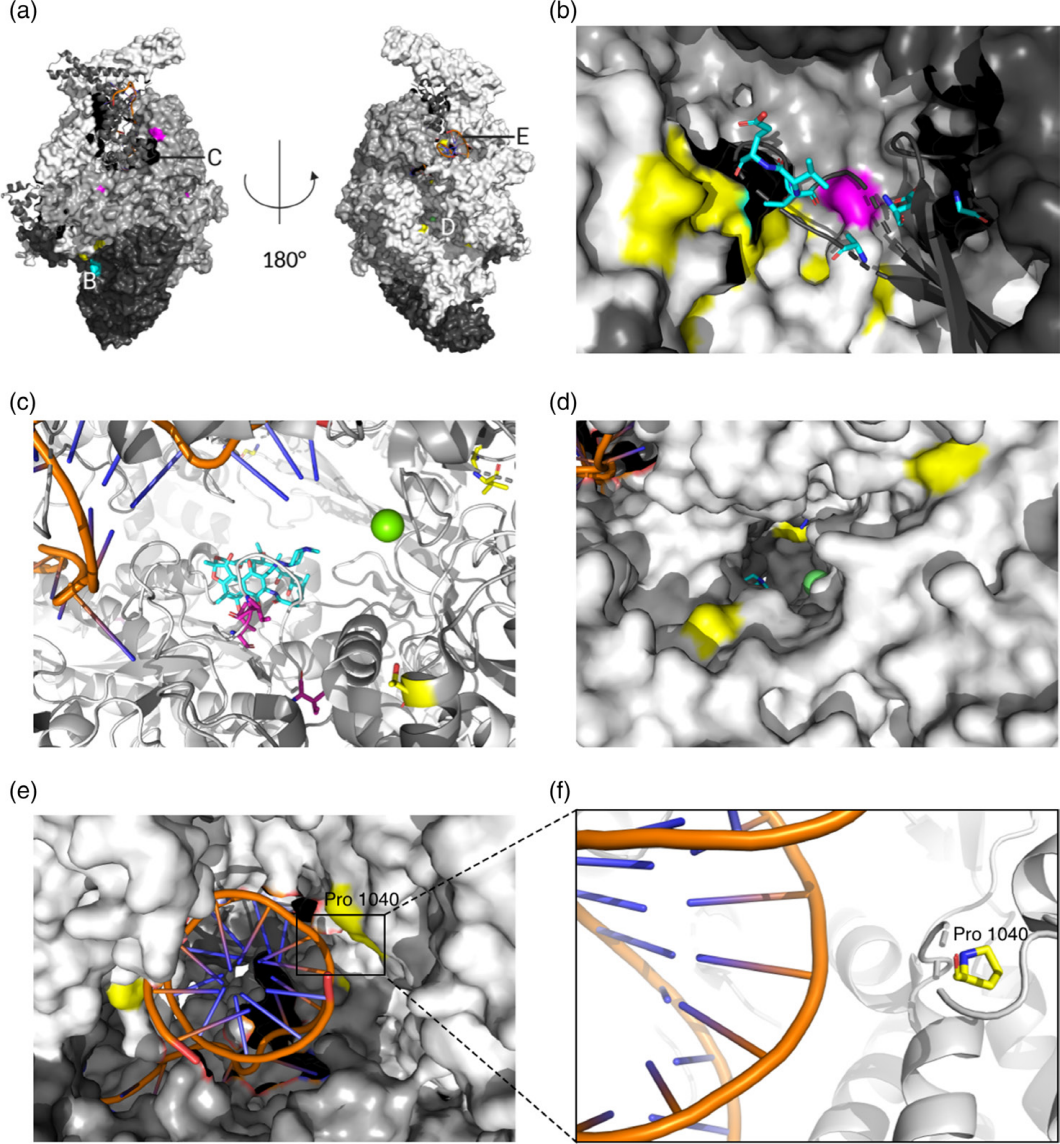
Compensatory mutations (CMs) map to various subunits of the RNA polymerase (RNAP). (**a**) Overview of clustering regions for CMs. Letters indicate where the CM clustering regions are located. (**b**) The interaction region of subunits α (black), β (dark grey) and β′ (light grey). CMs can be found in all these subunits and are highlighted in colour (β subunit, magenta; β′ subunit, yellow; α subunit, light blue, stick representation). (**c**) CMs close to the rifampicin resistance-determining region (RRDR) on the β and β′ subunits. Rifampicin (RIF, light blue) is shown bound to the RRDR. The DNA strand is visible on the top left in dark blue and orange stick representation. The active centre magnesium ion is shown in green. CMs are highlighted in colour (β subunit, magenta and β′ subunit, yellow) and by stick representation. (**d**) CMs close to the RNAP secondary channel in the β′ subunit (light grey) are shown in yellow. The location of the active site inside the protein can be deduced through the active site magnesium ion, indicated in green. (**e**) CMs close to the DNA entry channel are shown in yellow. The DNA helix is shown in dark blue and orange stick representation. (**f**) Close-up of the location of a putative CM (yellow stick representation, CM mutates proline to arginine) close to the DNA backbone. This CM might change interactions of the RNAP with the DNA strand.

To evaluate their position on the protein structure, we mapped all 51 non-synonymous putative CMs that show homoplasy (Table S3) onto the RNAP in complex with RIF [14]. The hits clustered in four different regions of the RNAP (Fig. 6a): (i) at the interfaces between subunits (Fig. 6b), (ii) on the β and β′ subunits close to the RRDR and the active centre (Fig. 6c), (iii) around the secondary channel in the β′ subunit (Fig. 6d) and (iv) at the DNA entry channel (Fig. 6e). There were putative CMs in most subunits of the RNAP, but only those in the *rpoA*, *rpoB* and *rpoC* genes show homoplasy (Fig. 3b, Table S3) and most of them were found on the β′ (*rpoC* gene) subunit.

The majority of CMs, including those most common in our data set (Table S3: V483G/A and I491V/T), were located close to the interface of the β, β′ and α RNAP subunits (Figs 6b and S3). Several hits also clustered close to the RRDR, where RIF binds to the RNAP of susceptible bacteria (Fig. 6c). The RNAP secondary channel (Fig. 6d) and the DNA entry channel (Fig. 6e) are locations for CMs that, to our knowledge, have not been discussed in the literature. The latter is especially interesting, since we observed CMs very close to where the DNA strand enters the protein (Fig. 6f).

## Discussion

In this study, we have identified a comprehensive set of high-confidence compensatory mutations (CMs) in *M. tuberculosis* using our Genetics data set of 77 860 samples and also captured and dissected their influence on *in vitro* growth of the bacteria using our Growth data set comprising 13 990 samples. Overall, we identified 78 unique hits, of which 51 exhibit homoplasy (Table S3) and 12 hits that are, to our knowledge, novel. Through mapping these high-confidence putative CMs on the RNAP structure, we identified four CM clustering regions (Fig. 6a). Two of them have, to our knowledge, not been described before: the DNA entry tunnel and the RNAP secondary channel.

The fitness cost of rifampicin (RIF) resistance described previously by Gagneux *et al.* [20] is reflected in the growth distributions of samples with resistance versus samples with no resistance mutations (Fig. 2a), i.e. we observed that the reduced fitness in samples with resistance mutations is recapitulated by our *in vitro* growth data. Concerning the impact of CMs on growth, our findings indicate that while CMs increase *in vitro* growth to a certain extent, they can be associated with and/or may act synergistically with other growth-enhancing factors, which can be clade-specific, that boost growth yet further.

Their strong association with high-fitness clades makes CMs interesting candidates for further studies. There are two possible scenarios. Firstly it is possible that CMs may have been preceded by evolutionarily older secondary growth-associated mutations that push growth above wild-type levels. This is supported by the fact that the high-growth CM clusters were located in sublineages 2.2 and 2.2.7, respectively, which are mostly part of the modern Beijing sublineages. These lineages are known to exhibit increased virulence [44]. Secondly, it is possible that by partially restoring fitness after resistance acquisition, CMs could have facilitated the emergence of further mutations and hence the establishment of these high-fitness clades. This would make CMs a key determinant in the increased transmissibility observed in lineage 2. In both scenarios, the notion that CMs play an important role in the spread of the increasingly virulent lineage 2 is supported by the fact that this lineage accumulated CMs more than any other lineage, presumably due to the reported higher mutation rates [45]. It has been suggested before that such compensated multidrug-resistant (MDR) mutants might be selected for in environments where many patients are treated with antituberculosis drugs [44]. This emphasizes the need for appropriate antibiotic stewardship to prevent further spread of MDR *M. tuberculosis*.

CMs are likely to exert different effects, depending on their location in the protein structure. The secondary channel serves as a direct connection from the outside of the protein to the active centre and is presumed to facilitate the diffusion of substrate nucleotides into the protein for incorporation into the nascent mRNA. It has been proposed that molecules entering through this channel could regulate RNAP activity as well [46]. CMs at this location could therefore modify diffusion in and out of the RNAP. The other novel location with a CM cluster was the DNA entry channel, and mutated residues in there could alter interactions with the DNA helix. However, most of the conformational changes caused by CMs affect the interface between the subunits and the space around the active site. CMs in the interfacial region might alter binding of the subunits, perhaps leading to higher protein stability, without necessarily affecting the active site [32]. Mutations close to the RRDR have been suspected to alter the conformation of the active site, possibly leading to higher activity of the enzyme [31]. Most CMs are hence likely to enhance transcriptional activity of the RNAP either through changing the conformation of the active site or through increasing the overall stability of the protein complex. The increased activity could, in turn, lead to increased transmissibility. Whether CMs are directly associated with transmission rates is an ongoing discussion in the field [47–52], with most sources supporting the notion of CMs being associated with increased transmission. Recent evidence even shows that *in vivo* fitness might be enhanced by compensatory evolution, thereby complementing our *in vitro* results [53]. Our results support a positive influence of CMs on growth and, consequently, on fitness in samples with CMs in lineage 2. This could translate to higher transmission rates at population level, although this has to be confirmed directly through *in vivo* experiments.

The nature of our growth data is a general limitation of our approach to capturing the fitness effects of CMs. The photographs taken by the Vizion instrument had moderate resolution and occasionally were affected by shadows and other artefacts, such as condensation [33]. In addition, due to uncertainty in algorithmically locating the wells, growth is only measured in the centre of each well. Taken together, the measured percentage growth is therefore somewhat noisy and should only be compared between large number of samples, as here. Also, because growth was measured after 2 weeks of incubation, any information on fitness advantages visible during the exponential growth phase, such as growth rate, will be lost. This could be changed by including growth monitoring during the exponential growth phase of the bacteria. Since this is difficult for a slow-growing species like *M. tuberculosis*, polymerase activity assays could potentially close this gap. By testing laboratory-derived *M. tuberculosis* mutants with an engineered combination of resistance mutations and CMs, the direct influence of CMs on RNAP activity could be tested. Even so, as the growth data were acquired *in vitro*, any fitness advantages that arise from the interaction of *M. tuberculosis* bacteria with their host environment will not be captured. We hence cannot necessarily directly translate the results of this study to the epidemiological reality of *M. tuberculosis* spread in human populations. As this was outside the scope of this study, we also did not account for possible associations between mutations outside of the RNA polymerase (RNAP) and RIF resistance. One could, however, adapt our statistical association testing method for other genes. On a similar note, it would be possible to model the growth data using a regression model, which would permit the inclusion of known confounding factors as variables to explain the observed phenotypes. This could help disentangle the lineage and clade associations of our high-growth phenotypes. However, this is beyond the scope of this paper.

Overall, the whole-genome sequencing data enabled the construction of a tractable, high-confidence list of 51 putative CMs, which could form the basis of future investigations. In addition, we derived further insights by combining the sequencing data with our *in vitro* growth data, allowing us to estimate the changes of the growth phenotype following emergence of resistance and CMs. CMs arise in a similar fashion in other *M. tuberculosis* genes, such as *ahpC* and *gyrA*, following resistance to isoniazid and fluoroquinolones, respectively [54]. Even in other bacterial species, such as *Salmonella typhimurium* and *Escherichia coli*, there is evidence for a fitness cost due to antibiotic resistance and compensatory mechanisms arising to reduce its impact [55]. Our approach to identifying CMs could hence be applied in a similar fashion to other micro-organisms and drugs where resistance mutations are annotated and known to introduce a fitness cost.

## Supplementary Data

Supplementary material 1
