# Supplementary material for: Compensatory mutations are associated with increased in vitro growth in resistant clinical samples of Mycobacterium tuberculosis
Source: Microb Genom. 2024 Feb 5;10(2):001187. doi: 10.1099/mgen.0.001187 (PMC10926696; doi:10.1099/mgen.0.001187)
Supplement: Supplementary material 1 [file mgen-10-1187-s001.pdf]

Compensatory mutations are associated with increased *in vitro* growth in resistant clinical samples of *Mycobacterium tuberculosis* – Supplemental Information

Viktoria Brunner[1] and Philip W Fowler*[1,2,3]

[1]Nuffield Department of Medicine, University of Oxford, Oxford, UK

[2]National Institute of Health Research Oxford Biomedical Research Centre, John Radcliffe Hospital, Headley Way, Oxford, UK

[3]Health Protection Research Unit in Healthcare Associated Infections and Antimicrobial Resistance, University of Oxford, UK

mutation	median growth [%]	p_{S450L}	p_{H445Y}	p_{D435V}	n
rpoB S450L	16.7		0.4193	0.2664	196
rpoB H445Y	15.5	0.4193		0.6445	42
rpoB D435V	16.4	0.2664	0.6445		325

Table S1: Median growth of resistant samples with specific resistance mutations. Mann-Whitney p-values are given with respect to the subscripted sample type and n indicates the sample size. Growth distributions of these samples are shown in Figure 2.

*To whom correspondence should be addressed: philip.fowler@ndm.ox.ac.uk, @philipwfowler

putative CM	Exp. evidence	1	2	3	4	5	6	7	8	sum(ref)	fisher
rpoC V483G	✓	✓	✓	✓	✓	✓	✓	✓		7	✓
rpoC V483A	✓	✓	✓	✓	✓			✓		5	✓
rpoC I491T		✓	✓	✓		✓	✓			5	✓
rpoC L527V		✓	✓		✓	✓				4	✓
rpoA T187A		✓	✓			✓			✓	4	✓
rpoC G332R				✓	✓	✓		✓		4	✓
rpoC L507V			✓		✓		✓	✓		4	
rpoC N698S		✓	✓	✓	✓					4	✓
rpoC W484G		✓	✓						✓	3	✓
rpoC I491V	✓	✓	✓		✓					3	✓
rpoC V517L		✓	✓		✓					3	✓
rpoC L516P	✓	✓			✓			✓		3	✓
rpoC H525Q			✓	✓	✓					3	
rpoC N698K	✓	✓			✓		✓			3	✓
rpoC V1252L	✓				✓		✓	✓		3	✓
rpoC F452L	✓						✓			1	✓
rpoC P1040R	✓				✓					1	✓

Table S2: Reference CMs used for evaluating our approach to identifying new CMs. Putative CMs are shown on the left. We included reference CMs that were either proven experimentally or identified in at least three of the reference papers. Fisher indicates if the CM came up as significantly resistance associated in our statistical association test and was located below the heuristic p-value threshold and showed homoplasy.

resistance	putative CM	only CM	both	$-\log_{10}$(p-value)	literature evidence	homoplasy
rpoB S450L	*rpoC* E1092D	2012	1989	inf		
rpoB S450L	*rpoC* V483G	37	1206	inf	✓	✓
rpoB S450L	*rpoC* I491V	19	665	inf	✓	✓
rpoB S450L	*rpoC* V483A	33	586	inf	✓	✓
rpoB S450L	*rpoC* I491T	10	457	293.06	✓	✓
rpoB S450L	*rpoC* P1040R	32	396	225.09	✓	✓
rpoB S450L	*rpoC* F452S	2	345	230.58	✓	✓
rpoB S450L	*rpoB* E761D	0	304	207.05		
rpoB S450L	*rpoB* L731P	1	226	151.44	✓	✓
rpoB S450L	*rpoC* N698S	2	205	135.23	✓	✓
rpoB S450L	*rpoC* D485Y	8	194	118.89	✓	✓
rpoB S450L	*rpoC* V517L	1	184	122.87	✓	✓
rpoB S450L	*rpoC* G332S	16	179	100.19		✓
rpoB S450L	*rpoC* V1252L	3	175	113.24	✓	✓
rpoB S450L	*rpoA* T187A	3	171	110.54	✓	✓
rpoB S450L	*rpoC* D485N	2	166	108.82	✓	✓
rpoB S450L	*rpoC* L516P	3	144	92.37	✓	✓
rpoB S450L	*rpoC* G433S	3	141	90.35	✓	✓
rpoB S450L	*rpoB* R827C	8	123	72.06		✓
rpoB S450L	*rpoC* P1040S	3	118	74.93		✓
rpoB S450L	*rpoC* L527V	3	113	71.58	✓	✓
rpoB S450L	*rpoC* G332R	5	113	68.95	✓	✓
rpoB S450L	*rpoC* P1040A	1	110	72.70	✓	✓
rpoB L452P	*rpoB* I1106T	3	103	212.49		
rpoB D435G	*rpoB* I1106T	3	103	263.70		
rpoB S450L	*rpoC* K445R	0	98	66.49		✓
rpoB S450L	*rpoC* F452L	3	96	60.24	✓	✓
rpoB S450L	*rpoC* L547V	2	80	50.94		
rpoB S450L	*rpoC* W484G	11	80	41.69	✓	✓
rpoB S450L	*rpoB* A692T	16	79	37.45		
rpoB S450L	*rpoB* I480V	3	78	48.27	✓	✓
rpoB S450L	*rpoA* V183G	10	77	40.62	✓	✓
rpoB S450L	*rpoB* K891E	0	74	50.18		✓
rpoB S450L	*rpoC* N416S	3	72	44.30	✓	✓
rpoB S450L	*rpoB* Q409R	14	69	32.77		✓
rpoB S450L	*rpoC* V1039A	1	68	44.37		✓
rpoB S450L	*rpoB* P45S	5	65	37.49	✓	✓
rpoB S450L	*rpoB* Q975H	17	65	28.57		
rpoB S450L	*rpoC* A521D	2	65	40.93	✓	✓
rpoB S450L	*rpoC* L507V	1	64	41.68	✓	✓
rpoB S450L	*rpoC* N826T	0	64	43.39		
rpoB S450L	*rpoC* V431M	4	62	36.58	✓	✓
rpoB S450L	rpoB V496A	0	60	40.68		
rpoB S450L	*rpoB* I488V	2	59	36.94		✓
rpoB S450L	rpoC V1252M	2	59	36.94	✓	✓
rpoB S450L	*rpoB* A286V	4	56	32.68	✓	✓
rpoB S450L	*rpoC* T812I	3	51	30.48	✓	✓
rpoB S450L	*rpoC* K1152Q	0	51	34.57		
rpoB S450L	*rpoB* R827L	0	50	33.89	✓	✓
rpoB S450L	*rpoC* L449V	0	50	33.89	✓	✓

rpoB S450L	*rpoC* N698K	4	48	27.50	✓	✓
rpoB S450L	*rpoC* F452C	1	48	30.94	✓	✓
rpoB S450L	*rpoA* A180V	0	48	32.54		✓
rpoB I491F	*rpoC* E1033A	25	46	95.60		✓
rpoB S450L	*rpoA* D190G	0	46	31.18	✓	✓
rpoB S450L	*rpoA* G31S	0	44	29.82	✓	✓
rpoB S450L	*rpoC* P434R	3	44	25.91	✓	✓
rpoB S450L	*rpoA* E184D	0	40	27.11		✓
rpoB H445R	*rpoC* S561P	2	39	98.27	✓	✓
rpoB V170F	*rpoB* V168A	2	25	64.98		
rpoB L452P	*rpoB* H1028R	1	23	46.47		
rpoB S450W	*rpoA* P25R	1	20	45.52		
rpoB H445D	*rpoC* G388A	1	17	32.33	✓	✓
rpoB D435G	*rpoB* I491L	18	17	32.89		✓
rpoB D435Y	*rpoB* R167C	1	15	29.72		
rpoB S450W	*sigA* A223T	0	15	35.07		
rpoB H445Y	*rpoB* E207K	0	15	29.05		
rpoB V170F	*rpoC* G571R	11	14	30.91	✓	
rpoB S441A	*rpoC* L405M	28	12	36.05		
rpoB S441A	*rpoB* L464M	3	11	39.12		
rpoB Q432P	*rpoC* T853A	3	10	28.72		
rpoB Q432K	*rpoZ* T107I	1	10	30.13		
rpoB S441A	*sigA* E385Q	14	9	27.77		
rpoB S441A	*sigA* G380A	3	9	31.34		
rpoB S441A	*sigA* I382V	3	9	31.34		
rpoB S441A	*sigA* L386M	4	9	30.83		
rpoB S441A	*rpoB* E460D	5	8	26.66		
rpoB S441A	*rpoC* I128V	3	8	27.56		
rpoB S441A	*rpoB* R791T	0	7	25.92		

Table S3: Hit list resulting from Fisher's exact test for association of resistance with co-occuring mutations, after removing synonymous mutations. The first column indicates the resistance mutation that the putative compensatory mutation (CM) in the second column is associated to. 'Only CM' indicates how often the CM occurs on its own, without the corresponding resistance mutation, and 'both' indicates how often we see the two mutations occur together. The last two columns indicate if the CM has been mentioned in the literature and if it shows homoplasy, respectively.

sample type	median growth [%]	CI low	CI high	p-value$_r$	p-value$_s$	n
pan-susceptible	22.1	21.6	22.7			5283
resistant and no CMs	18.7	18.0	19.3		3.92e-15	2869
resistant and CMs	26.6	25.5	27.4	3.92e-57	6.25e-26	2667

Table S4: Median growth of resistant samples with compensatory mutations compared to pan-susceptible samples and samples with only resistance mutations. The confidence interval (CI) for the median is calculated using bootstrapping where 'CI low' indicates the lower threshold and 'CI high' the upper threshold. P-values are given with respect to resistant (p-value$_r$) and pan-susceptible sample growth (p-value$_s$) and n indicates the sample size.

Lineage	median growth [%]	CI low	CI high	p-value$_1$	p-value$_2$	p-value$_3$	n
Lineage 1	23.1	20.6	25.5				534
Lineage 2	26.1	25.3	27.0	7.17e-02			1331
Lineage 3	32.1	29.6	34.8	8.68e-14	8.36e-16		706
Lineage 4	18.1	17.6	18.6	1.22e-05	7.99e-33	3.31e-64	2656

Table S5: Median growth of pan-susceptible samples from different *M. tuberculosis* lineages. The confidence interval (CI) for the median is calculated using bootstrapping where 'CI low' indicates the lower threshold and 'CI high' the upper threshold. P-values are given with respect to each lineage, indicated by the subscript x (p-value$_x$) and n indicates the sample size.

sample type	median growth [%]	CI low	CI high	p-value$_r$	p-value$_s$	n
Lineage 1:						
pan-susceptible	23.1	20.6	25.3			534
resistant and no CMs	22.9	19.6	28.7		0.784	126
resistant and CMs	23.3	19.6	25.2	0.516	0.596	58
Lineage 2:						
pan-susceptible	26.1	25.4	27.2			1331
resistant and no CMs	21.6	20.4	22.5		2.46e-05	1103
resistant and CMs	31.4	30.4	32.5	5.65e-43	3.20e-24	1788
Lineage 3:						
pan-susceptible	32.1	29.6	34.9			706
resistant and no CMs	24.4	22.4	27.2		8.79e-09	370
resistant and CMs	26.3	23.7	33.2	2.07e-02	4.38e-02	190
Lineage 4:						
pan-susceptible	18.1	17.6	18.6			2656
resistant and no CMs	14.4	13.7	15.2		6.17e-17	1252
resistant and CMs	14.6	13.7	15.4	0.995	3.44e-11	626

Table S6: Lineage-wise median growth of samples with different compensatory mutations compared to pan-susceptibles and samples with only resistance. The confidence interval (CI) for the median is calculated using bootstrapping where 'CI low' indicates the lower threshold and 'CI high' the upper threshold. P-values are given with respect to resistant (p-value$_r$) and pan-susceptible sample growth (p-value$_s$) and n indicates the sample size.

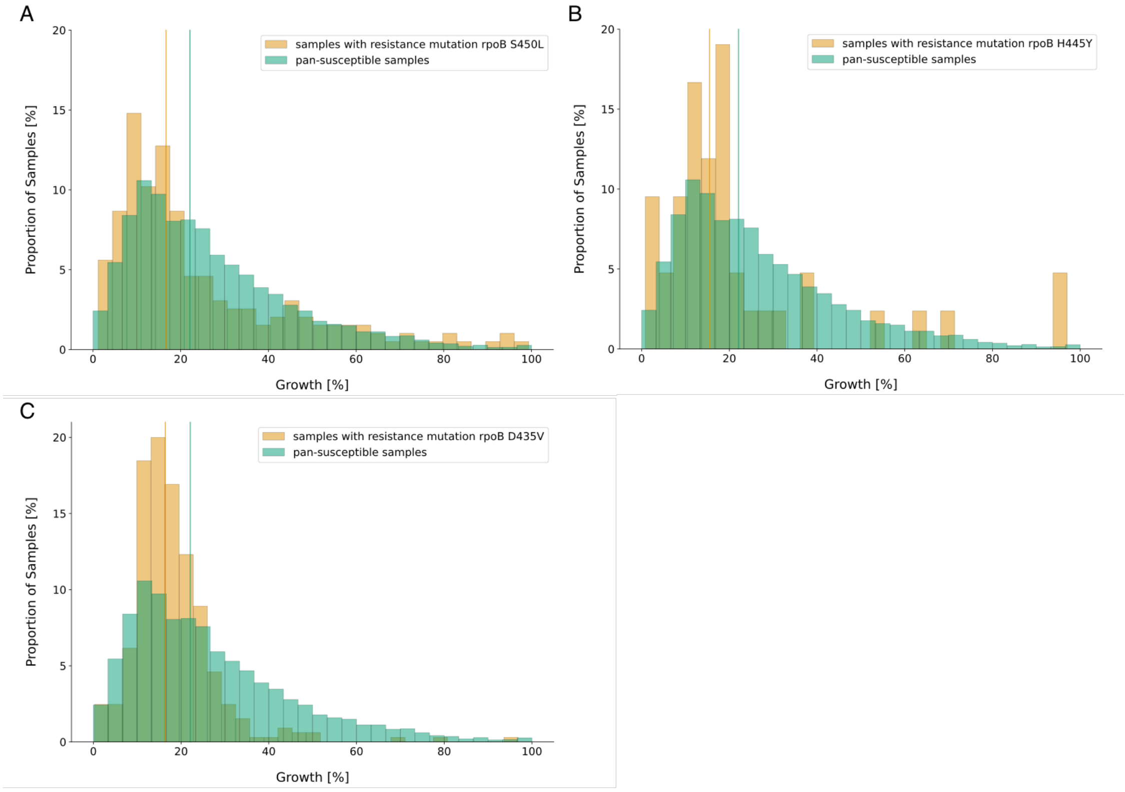

Figure S1: Growth distributions for pan-susceptible samples vs samples with specific rifampicin (RIF) resistance mutations in *M. tuberculosis* (A-C) Distributions of growth in percent of covered well-area as measured in the CRyPTIC project[9] were plotted as a histogram against the proportion of samples that display this amount of growth. Samples with the resistance mutation indicated in the legend and no other potentially interfering mutations are plotted in red, samples that were classified as pan-susceptible are plotted in green. Vertical lines indicate the respective medians. The medians and Mann-Whitney p-values of the distributions are listed in Table 1.

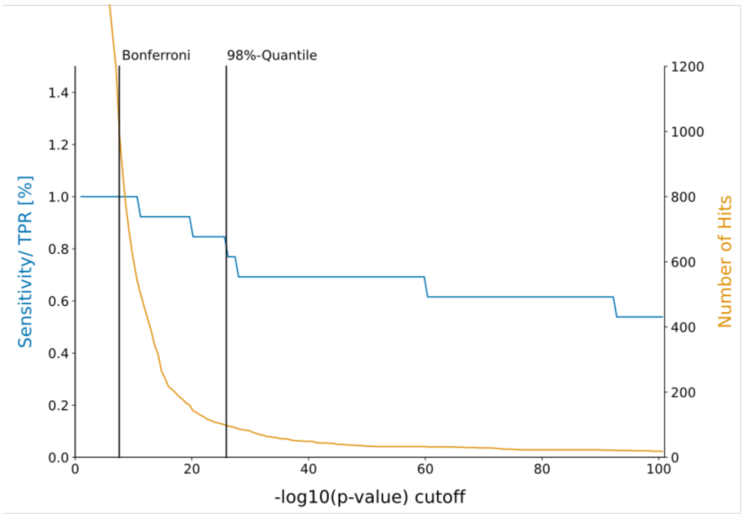

Figure S2: Sensitivity and number of significant hits (putative compensatory mutations) depending on p-value The graph shows the number of significant hits and reference hits detected depending on the $\log_{10}$ p-value cutoff shown on the x-axis. The left y-axis refers to the percentage of found reference hits from a compiled list, also termed sensitivity or true positive rate (TPR). The right y-axis shows the number of mutations that were classified as significantly resistance associated under the respective cut-off. The vertical lines indicate the p-value cut-off with Bonferroni correction and our heuristic p-value cut-off at the 98% quantile, respectively.

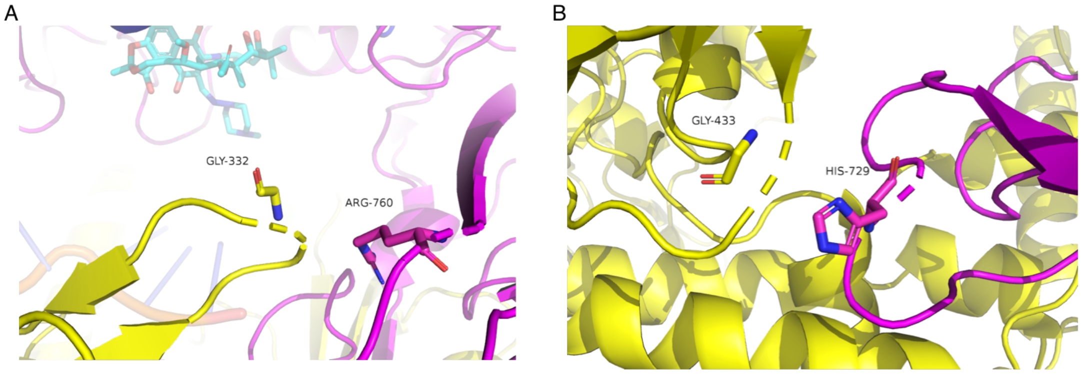

Figure S3: Location of two high-confidence compensatory mutations (CMs) on the RNA polymerase (RNAP) (A) The CM G332S is located on the β' subunit, in a contact region to the β subunit (magenta). The change from Glycine (stick representation) to Serine (negatively charged side chain) might enable an interaction with the close-by Arginine (positively charged side chain) on the β subunit. The bound drug rifampicin (light blue) can be seen in the background. **(B)** The CM G433S is located on the β' subunit, in a contact region to the β subunit. The change from Glycine (stick representation) to Serine might enable an interaction with the close-by Histidine (positively charged side chain) on the β subunit.

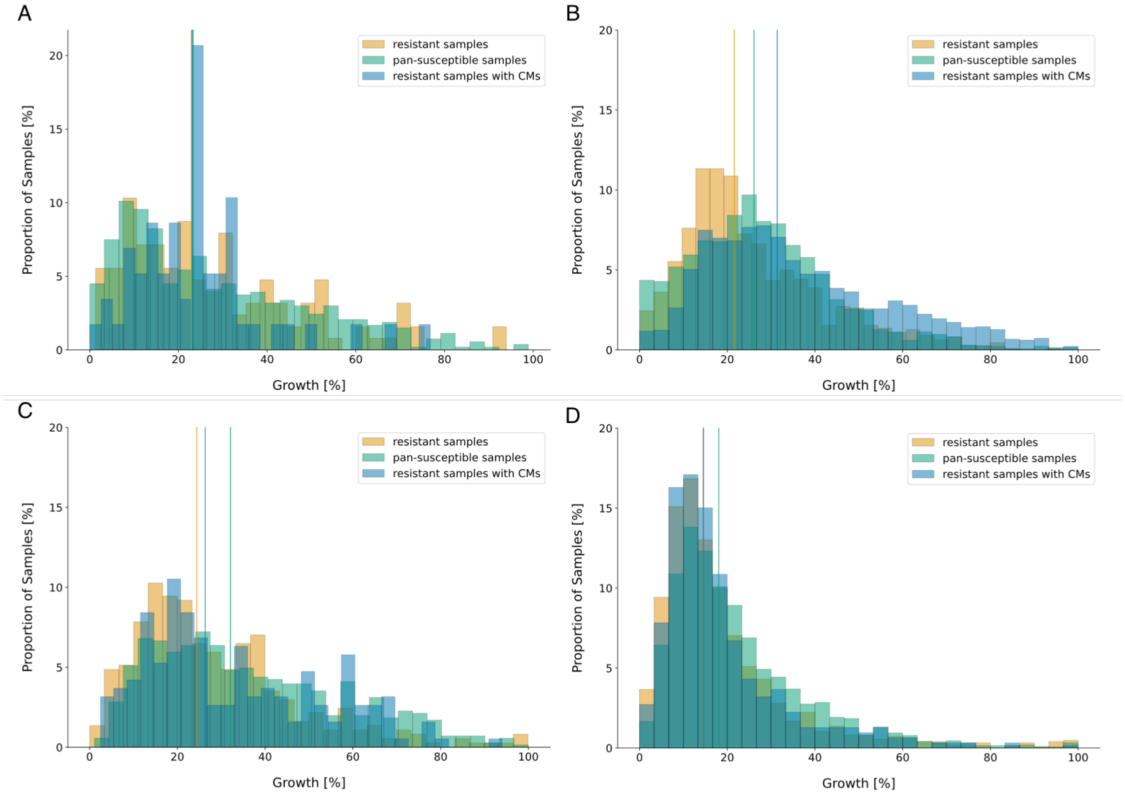

Figure S4: Growth distributions of *M. tuberculosis* samples within different lineages (A) Distribution of growth in *M. tuberculosis* Lineage 1 in percent of covered well-area as measured in the CRyPTIC project[9] were plotted as a histogram against the proportion of samples that display this amount of growth. Samples with rifampicin (RIF) resistance mutations but no putative compensatory mutations (CMs) are plotted in red, samples that were classified as pan-susceptible are plotted in green. Samples that have RIF resistance mutations and at least one CM are shown in blue. Vertical lines indicate the respective medians. The medians and Mann-Whitney p-values of the distributions are shown in Supplementary Table S5. **(B)** Plot layout as in (A), but samples derive from *M. tuberculosis* Lineage 2. **(C)** Plot layout as in (A), but samples derive from *M. tuberculosis* Lineage 3. **(D)** Plot layout as in (A), but samples derive from *M. tuberculosis* Lineage 4.

References

[1] I. Comas, S. Borrell, A. Roetzer, et al. (2012) Whole-genome sequencing of rifampicin-resistant Mycobacterium tuberculosis strains identifies compensatory mutations in RNA polymerase genes, *Nature Genetics* 44:106–110.

[2] Q. Li, W. Jiao, Q. Yin, et al. Compensatory Mutations of Rifampin Resistance Are Associated with Transmission of Multidrug-Resistant Mycobacterium tuberculosis Beijing Genotype Strains in China, *Antimicrobial Agents and Chemotherapy* 60:2807–2812.

[3] M. de Vos, B. Müller, S. Borrell, et al. (2013) Putative Compensatory Mutations in the rpoC Gene of Rifampin-Resistant Mycobacterium tuberculosis Are Associated with Ongoing Transmission, *Antimicrobial Agents and Chemotherapy* 57:827–832.

[4] V. Ruiz and A. Paula (2020) Determination of potentially novel compensatory mutations in rpoC associated with rifampin resistance and rpoB mutations in Mycobacterium tuberculosis clinical isolates from Peru, *Int J Mycobacteriol* 9:121–137.

[5] N. Casali, V. Nikolayevskyy, Y. Balabanova, et al. (2012) Microevolution of extensively drug-resistant tuberculosis in Russia, *Genome Research* 22:735–745.

[6] T. Song, Y. Park, I. C. Shamputa, et al. (2014) Fitness costs of rifampicin resistance in Mycobacterium tuberculosis are amplified under conditions of nutrient starvation and compensated by mutation in the subunit of RNA polymerase, *Molecular Microbiology* 91:1106–1119.

[7] A. Ali, Z. Hasan, R. McNerney, et al. (2015) Whole Genome Sequencing Based Characterization of Extensively Drug-Resistant Mycobacterium tuberculosis Isolates from Pakistan, *PLOS ONE* 10:e0117771.

[8] P. Ma, T. Luo, L. Ge, et al. (2021) Compensatory effects of M. tuberculosis rpoB mutations outside the rifampicin resistance-determining region, *Emerging Microbes & Infections* 10:743–752.

[9] P. W. Fowler, A. L. Gibertoni Cruz, S. J. Hoosdally, et al. (2018) Automated detection of bacterial growth on 96-well plates for high-throughput drug susceptibility testing of Mycobacterium tuberculosis, *Microbiology* 164:1522–1530.
